# Anti-Virulence and Antioxidant Activities of *Weissella confusa* WM36 Supernatant Against *Salmonella *Typhimurium

**DOI:** 10.3390/microorganisms14061321

**Published:** 2026-06-12

**Authors:** Wattana Pelyuntha, Netnapa Makhamrueang, Sasithorn Sirilun

**Affiliations:** 1 Futuristic Science Research Center, School of Science, Walailak University, Thasala, Nakhon Si Thammarat 80160, Thailand; wattana.pe@wu.ac.th; 2 Research Center for Theoretical Simulation and Applied Research in Bioscience and Sensing, Walailak University, Thasala, Nakhon Si Thammarat 80160, Thailand; 3 Department of Pharmaceutical Sciences, Faculty of Pharmacy, Chiang Mai University, Chiang Mai 50200, Thailand; netnapa.ma@cmu.ac.th; 4 Office of Research Administration, Chiang Mai University, Chiang Mai 50200, Thailand; 5 Innovation Center for Holistic Health, Nutraceuticals, and Cosmeceuticals, Faculty of Pharmacy, Chiang Mai University, Chiang Mai 50200, Thailand

**Keywords:** biofilm, cell-free supernatant, *Salmonella*, postbiotics, *Weissella confusa*

## Abstract

Lactic acid bacteria (LAB) have been widely utilized in the production of fermented foods worldwide due to their well-established health-promoting benefits for both humans and animals. In addition to their nutritional value, LAB exhibit antagonistic activity against foodborne pathogens, particularly *Salmonella* spp., which are commonly associated with livestock and animal production systems. LAB exert a range of biological effects that can inhibit the growth of *Salmonella* and modulate its virulence. In the present study, the antagonistic potential of *Weissella confusa* WM36 was evaluated based on its ability to inhibit *S.* Typhimurium growth, disrupt biofilm formation, and suppress the expression of virulence-associated genes. A preliminary safety assessment of *W. confusa* WM36 was conducted through hemolytic activity and antibiotic susceptibility profiling. In addition, the biofunctional properties of its cell-free supernatant (CFS), herein referred to as postbiotic metabolites, were investigated with a particular focus on antioxidant activity. Experimental results demonstrated that *W. confusa* WM36 and its CFS at 40% (*v*/*v*) achieved a complete reduction (100%) of *S. *Typhimurium cell counts within 6 to 12 h of treatment. Furthermore, CFS at 20% and 40% (*v*/*v*) significantly impaired biofilm formation, while treatment with 20% (*v*/*v*) CFS markedly downregulated the expression of key virulence genes. The strain WM36 exhibited α-hemolytic activity and showed susceptibility to most of the antibiotics tested, although resistance to ceftriaxone and trimethoprim–sulfamethoxazole was observed. These findings provide preliminary information regarding its safety characteristics; however, further molecular and in vivo investigations are required to comprehensively evaluate its safety for practical applications. Additionally, the CFS exhibited notable antioxidant activity, with DPPH radical scavenging capacity of 8.90 ± 0.06 mM Trolox equivalents and ABTS radical scavenging power of 13.10 ± 1.42 mM Trolox equivalents. Collectively, these findings highlight the potential of *W. confusa* WM36 and its postbiotic metabolites as promising biocontrol and functional agents against *S. *Typhimurium, while further safety validation remains necessary.

## 1. Introduction

Lactic acid bacteria (LAB) are among the most extensively utilized microbial groups in the production of a wide range of fermented foods, including dairy products, fruits and vegetables, meats, and more. Their application is largely attributed to their functional properties, which confer various health and food preservation benefits. LAB possess unique metabolic characteristics, notably the production of organic acids, which acidify the environment, thereby extending the shelf life of food products and inhibiting the growth of spoilage microorganisms [1]. Moreover, LAB exert potential health benefits for both humans and animals. For instance, they can mitigate infections caused by foodborne pathogens and urinary tract infections through the production of antimicrobial compounds. Probiotic LAB also play an important role in modulating gut microbiota and enhancing host immune responses [2,3]. LAB are Gram-positive, acid-tolerant, non-spore-forming bacteria, with morphologies ranging from rod-shaped to spherical. The genera include *Lactobacillus*, *Lactococcus*, *Leuconostoc*, *Pediococcus*, *Streptococcus*, *Aerococcus*, *Carnobacterium*, *Enterococcus*, *Oenococcus*, *Sporolactobacillus*, *Tetragenococcus*, *Vagococcus,* and *Weissella*. Additionally, species from the genus *Bifidobacterium* are often associated with LAB due to similar probiotic functions [4,5]. The primary metabolites produced by LAB are lactic acid and other organic acids derived from carbohydrate catabolism. Certain LAB strains are also capable of producing bacteriocins, antimicrobial peptides that serve as secondary metabolites with potent bactericidal and bacteriostatic activities against various pathogenic and spoilage microorganisms [6].

*Weissella confusa* (*W. confusa*) (formerly *Lactobacillus confusus*) is a member of the LAB group, classified under the order *Lactobacillales* and the family *Leuconostocaceae*. Some isolates of *W. confusa* exhibit promising probiotic properties, particularly due to their antimicrobial activities. However, this species has also been implicated in opportunistic infections, primarily in immunocompromised hosts [7,8]. Despite these concerns, *W. confusa* plays a functional role in food fermentation and has been suggested as a potential probiotic [9]. Nevertheless, the application of *W. confusa* as a probiotic or biocontrol agent requires careful safety consideration. Although many isolates have been recovered from fermented foods and healthy microbiota, several clinical cases have reported *W. confusa* as an opportunistic pathogen associated with bacteremia, endocarditis, and other invasive infections, particularly in immunocompromised individuals [7,8]. In addition, some strains exhibit intrinsic resistance to certain antibiotics, including vancomycin, raising concerns regarding their safety and potential contribution to antimicrobial resistance dissemination. Consequently, the probiotic and biocontrol potential of *W. confusa* should be evaluated on a strain-specific basis, with comprehensive assessments of both beneficial properties and safety-related characteristics prior to practical application in food, animal, or human health settings. LAB have been widely investigated as alternative interventions for controlling microbial contamination and infection. Numerous studies have described the antagonistic mechanisms of LAB against pathogens, including the production of inhibitory compounds, competitive exclusion at adhesion sites, interference with pathogenic colonization, and immunomodulation [10]. For example, LAB isolates such as *Enterococcus faecium* CAU 7856, *Lactobacillus brevis* CAU 9567 and CAU 9967, and *Pediococcus acidilactici* CAU 9896, sourced from children’s feces, demonstrated antibacterial activity against uropathogenic *Escherichia coli* [11]. *Lactobacillus salivarius* and *L. fermentum* inhibited both the growth and biofilm formation of methicillin-resistant *Staphylococcus aureus* (MRSA) [12]. Similarly, strains including *L. delbrueckii*, *L. casei*, *L. curvatus*, *P. acidilactici,* and *Leuconostoc* spp. have shown antagonistic effects against *Listeria monocytogenes* isolated from contaminated frozen and ready-to-eat foods [13,14]. *Lactiplantibacillus plantarum* WPL10 showed a potential biocontrol agent by producing quorum quenching (QQ) compounds [15]. Furthermore, fifteen *Lactobacillus* species have demonstrated inhibitory activity against *Salmonella* strains isolated from cattle feces [16]. Notably, our two *Weissella* strains have been reported to suppress the growth, biofilm formation, and virulence of *S.* Typhi and *S. *Typhimurium [17,18]. Despite the growing interest in LAB for pathogen control, most studies have focused on genera other than *Weissella*. Our previous study investigated the antagonistic effects of *W. confusa* WM36 against *S.* Typhi [18]. It has been shown to produce antimicrobial compounds, including organic acids and 2,4-Di-*tert*-butylphenol (2,4-DTBP), which contribute to its inhibitory effects on *S.* Typhi [18].

*Salmonella* spp. are among the most significant foodborne bacteria, responsible for gastrointestinal illnesses and systemic infections collectively referred to as salmonellosis. Transmission primarily occurs via the fecal-oral route through the consumption of contaminated food and water [19].

Among the numerous serovars, *S. enterica* serovar Typhimurium is considered one of the most important zoonotic pathogens because of its broad host range, frequent association with livestock and poultry, and its prominent role in foodborne outbreaks worldwide [20,21,22]. This serovar is of particular concern due to its ability to colonize the intestinal tract, persist in food production environments, and spread through the food chain, thereby posing a substantial threat to both public health and animal production. In addition, the emergence of antimicrobial-resistant *S. *Typhimurium strains has further increased the clinical and epidemiological importance of this pathogen [23,24], as such strains may complicate treatment and contribute to more severe or prolonged infections. Its pathogenicity is largely attributed to the expression of genes located within specific *Salmonella* pathogenicity islands (SPIs) [25]. These SPIs regulate virulence gene expression and contribute to multiple infection mechanisms. SPI-1 encodes a type III secretion system (T3SS) responsible for bacterial invasion and modulation of host immune responses [25,26]. Other virulence factors, such as fimbriae, flagella, and biofilm production, also contribute to pathogenesis. Additionally, quorum sensing (QS) systems play a critical role in regulating both SPI-1 expression and biofilm development [27].

Therefore, the objectives of this study were to evaluate the antagonistic effects of *W. confusa* WM36 on the growth, biofilm formation, and virulence gene expression of *S. *Typhimurium. Both viable *W. confusa* WM36 cells and its CFS were investigated depending on the specific objective of each assay. The safety-related characteristics of WM36 were preliminarily assessed through hemolytic activity and antibiotic susceptibility testing. Furthermore, the antioxidant capacity of its CFS was examined. The findings from this study aim to expand current knowledge of *W. confusa* WM36 and its bioactive metabolites and support their potential for future development as biocontrol and functional agents against *Salmonella*.

## 2. Materials and Methods

### 2.1. Bacterial Strains and Growth Conditions

*S. enterica* serovar Typhimurium TISTR 1469 was obtained from the Department of Pharmaceutical Sciences, Faculty of Pharmacy, Chiang Mai University, Chiang Mai, Thailand. The strain was cultured in Tryptic Soy Broth (TSB) at 37 °C for 18 h. *W. confusa* WM36 was provided by the Innovation Center for Holistic Health, Nutraceuticals, and Cosmeceuticals, Faculty of Pharmacy, Chiang Mai University, and cultured in de Man, Rogosa, and Sharpe (MRS) medium at 37 °C for 18 h. This strain was previously isolated from fermented grapes in northern Thailand [17], was characterized as a Gram-positive, rod-shaped lactic acid bacterium. It is permanently deposited at the Thailand Bioresource Research Center (TBRC), Pathum Thani, Thailand, under the accession number TBRC 11086. All bacterial strains were reactivated and propagated in appropriate media and conditions prior to use in experimental analyses.

### 2.2. Preparation of Weissella confusa WM36 Cell-Free Supernatant

*W. confusa* WM36 was prepared by adjusting the cell concentration to approximately 6 log CFU/mL in fresh MRS broth. A 10 mL aliquot of the bacterial suspension was inoculated into 100 mL of MRS broth and incubated at 37 °C for 24 h. After incubation, the culture was centrifuged at 6000 rpm for 15 min at 4 °C to separate the cells. The resulting supernatant was then sterile-filtered through a 0.22 µm syringe filter and stored at −20 °C until further analysis.

### 2.3. Characterization of Antibacterial Substances of Weissella confusa WM36

The antimicrobial substances presented in *W. confusa* WM36-CFS against *S. *Typhimurium was preliminary characterized after pH, catalase, and proteinase K treatments using a 96-well plate assay. For pH treatment, the CFS was adjusted to pH 4.0, 5.0, and 6.0, while untreated CFS and sterile MRS broth served as controls. To determine the role of hydrogen peroxide, catalase (5 mg/mL final concentration) prepared in 0.05 mol/L phosphate buffer (pH 7.0) was added to the CFS and incubated at 37 °C for 2 h, followed by enzyme inactivation at 100 °C for 5 min before antimicrobial testing. Similarly, proteinase K treatment was conducted by adding the enzyme to a final concentration of 1 mg/mL, incubating at 37 °C for 2 h, heat-inactivating at 100 °C for 5 min prior to analysis. In all experiments, sterile MRS broth and untreated CFS (pH 6.0) were used as controls, and each condition was tested in triplicate.

### 2.4. Minimum and Sub-Minimum Effective Concentrations of Cell-Free Supernatant

To determine the MEC of *W. confusa* WM36-CFS against *S. *Typhimurium, a broth microdilution assay was conducted. Briefly, the CFS was serially diluted in TSB to obtain final concentrations ranging from 5% to 90% (*v*/*v*). An aliquot (10 µL) of *S. *Typhimurium inoculum (approximately 5 log CFU/mL) was then added to each well and incubated at 37 °C for 24 h. Bacterial growth was assessed by measuring turbidity at OD600 and comparing the values with those of the untreated control. The MEC was defined as the lowest concentration at which OD600 values were comparable to the sterile medium control [17,18].

### 2.5. Effect of Weissella confusa WM36 on Salmonella Typhimurium Growth

*W. confusa* WM36 and *S. *Typhimurium were pre-cultured separately under their respective optimal conditions. Each strain was adjusted to a final concentration of approximately 6–7 log CFU/mL in 10 mL of Luria Bertani (LB) broth. Equal volumes of each bacterial suspension were combined in a 100 mL Erlenmeyer flask and incubated at 37 °C. Samples were collected at 6 h intervals for 24 h. Serial ten-fold dilutions of the collected samples were prepared using 0.85% normal saline solution (NSS). For *W. confusa* enumeration, 1 mL of each diluted sample was poured into Petri dishes containing 20 mL of MRS agar (1.5% agar). For *S. *Typhimurium enumeration, 0.1 mL of the diluted sample was spread onto Xylose Lysine Deoxycholate (XLD) agar plates. Monocultures of each bacterium served as control groups.

To assess the effect of *W. confusa* WM36-CFS on *S. *Typhimurium growth, *S. *Typhimurium was cultured in 10 mL of LB broth supplemented with WM36-CFS at its previously determined minimum effective concentration. The control group consisted of *S. *Typhimurium cultured in LB broth without CFS supplementation. All plates were incubated at 37 °C for 24–48 h. The number of viable cells was enumerated and expressed as a percentage of bacterial survival (1) and reduction (2), calculated using the following formulas:

(1) % *W. confusa* survival = (No. of bacterial count in treatment / No. of bacterial count in control) × 100 …

(2) % *Salmonella* reduction = ((No. of bacterial count in control − No. of bacterial count in treatment) / No. of bacterial count in control) × 100 …

### 2.6. Effect of Weissella confusa WM36 on Salmonella Typhimurium Biofilm Formation

The effect of WM36-CFS on *Salmonella* biofilm formation was evaluated using a 24-well microplate containing a glass slide (15 × 8 mm) in each well. Two milliliters of LB broth supplemented with WM36-CFS at minimum and sub-minimum effective concentrations, along with a suspension of *S. *Typhimurium, were added to each well and incubated at 37 °C for 48 h. After incubation, all glass slides were stained with 0.4% crystal violet solution, washed three times with sterile distilled water, and air-dried at room temperature. The stained slides were examined under a stereomicroscope (Stemi 508, Carl Zeiss AG, Oberkochen, Germany) at 10× magnification. Visible biofilms were photographed using an attached digital camera (Axiocam 105 color, Zeiss, Germany) and analyzed with the ZEN 2(blue edition) software program (version 3.13.109.06000) [18].

### 2.7. Effect of Weissella confusa WM36 on Salmonella Typhimurium Virulence Gene Expression

The effect of WM36-CFS on *S. *Typhimurium virulence gene expression was investigated using real-time PCR analysis. Briefly, *S. *Typhimurium was cultured in LB broth supplemented with WM36-CFS at a sub-MEC of 20% (*v*/*v*). This concentration was selected because it did not completely inhibit bacterial growth, thereby allowing the evaluation of transcriptional responses in viable bacterial cells. In contrast, the MEC (40% *v*/*v*) resulted in complete growth inhibition and was therefore unsuitable for gene expression analysis. Bacterial cells were subsequently harvested by centrifugation at 6000 rpm for 15 min at 4 °C and adjusted to approximately 6 log CFU/mL.

Cells were treated with 1 mL of TRIzol^®^ reagent (Invitrogen, USA) and incubated for 5 min to dissociate nucleoprotein complexes. Subsequently, 0.2 mL of chloroform was added, followed by incubation for 3 min and centrifugation at 12,000× *g* for 15 min at 4 °C. The aqueous phase containing total RNA was transferred to a new tube, mixed with 0.5 mL of isopropanol, and incubated overnight at 4 °C. After incubation, samples were centrifuged at 12,000× *g* for 10 min at 4 °C. The resulting RNA pellet was visible as a white gel-like substance and the supernatant was carefully removed. The RNA pellet was washed with 0.5 mL of 75% ethanol, centrifuged at 7500× *g* for 5 min at 4 °C, and the supernatant was discarded. The pellet was air-dried for 15 min, then resuspended in 50 µL of nuclease-free water, heated at 60 °C for 15 min using a heat block, and stored at −20 °C until further analysis. Total RNA concentration was measured using a Qubit^®^ RNA BR Assay Kit (Life Technologies, USA) and a Qubit^®^ 3.0 Fluorometer. Complementary DNA (cDNA) was synthesized using the High-Capacity cDNA Reverse Transcription Kit (Applied Biosystems, USA). Each 20 µL reaction contained 10 µL of total RNA (0.5 µg), 2 µL of 10× RT buffer, 0.8 µL of 25× dNTP mix, 2 µL of 10× random primers, 1 µL of MultiScribe™ reverse transcriptase, 1 µL of RNase inhibitor, and 3.2 µL of nuclease-free water. The thermal cycler was programmed as follows: 25 °C for 10 min, 37 °C for 120 min, 85 °C for 5 min, and held at 4 °C. Real-time PCR was performed using a QuantStudio 6 Flex Real-Time PCR System (Thermo Fisher Scientific, MA, USA) under the following cycling conditions: initial activation at 50 °C for 2 min and 95 °C for 2 min, followed by 40 cycles of 95 °C for 20 s and 60 °C for 20 s. A melting curve was generated by heating to 95 °C for 20 s, cooling to 60 °C for 20 s, and ramping to 95 °C with 0.05 °C increments. Each 20 µL PCR reaction consisted of 2 µL of cDNA template, 10 µL of PowerUp™ SYBR^®^ Green Master Mix (Applied Biosystems™, MA, USA), 0.2 µL each of forward and reverse primers (Table 1), and 7.6 µL of nuclease-free water. The *Salmonella 16S rRNA* gene was used as an internal housekeeping control based on its previous application as a stable reference gene in studies evaluating *Salmonella* virulence gene expression under similar experimental conditions [18]. Gene expression levels were analyzed using the 2^−ΔΔCt^ method. Fold changes represent the expression of each target gene relative to the untreated control (reference sample), which was set to a fold change value of 1 [18,28,29].

### 2.8. Preliminary Safety Evaluation of Weissella confusa WM36

*W. confusa* WM36 was activated in 5 mL of fresh MRS broth and incubated at 37 °C overnight. The culture was then streaked onto the surface of a blood agar plate and incubated at 37 °C for 72 h. Subsequently, the plate was examined for the presence of hemolytic activity, indicated by the formation of clear (β-hemolysis), greenish (α-hemolysis), or no (γ-hemolysis) zones surrounding the colonies. Antibiotic susceptibility was assessed using the disc diffusion method. An overnight culture of *W. confusa* WM36 was evenly swabbed onto the surface of Mueller-Hinton Agar (MHA) supplemented with 5% sheep blood. Antibiotic discs were applied to the surface of the agar, including ampicillin (10 µg), azithromycin (15 µg), ceftriaxone (30 µg), chloramphenicol (30 µg), doxycycline (30 µg), erythromycin (15 µg), levofloxacin (5 µg), moxifloxacin (5 µg), penicillin (10 µg), tetracycline (30 µg), and trimethoprim–sulfamethoxazole (25 µg). The plates were incubated at 37 °C for 24 h. Inhibition zones around the discs were measured and interpreted with reference to the Clinical and Laboratory Standards Institute (CLSI) M45 guideline for fastidious organisms [30]. Because species-specific interpretive criteria for *Weissella* spp. are not currently available, the susceptibility classifications should be regarded as preliminary and interpreted with caution.

### 2.9. Antioxidant Activities of Weissella confusa WM36 Cell-Free Supernatant

The DPPH radical-scavenging activity of the WM36-CFS was determined as previously described by Herch et al. [31] and Widowati et al. [32], with slight modifications. Briefly, 100 µL of CFS was mixed with 145 µL of DPPH solution (Sigma-Aldrich, St. Louis, MO, USA) and incubated at room temperature for 30 min. The absorbance of the reaction mixture was measured at 515 nm using a microplate reader (SPECTROstar Omega, BMG LABTECH, Ortenberg, Germany). The DPPH radical-scavenging activity was calculated based on a standard curve of Trolox (Sigma-Aldrich, USA) ranging from 5 to 40 µM and expressed as the percentage of scavenging inhibition. All samples were analyzed in triplicate. The ABTS assay was performed following a modified protocol described by Tai et al. [33] and Jun et al. [34]. Briefly, 20 µL of CFS was mixed with 2 mL of 7 mM ABTS solution (Sigma-Aldrich, USA). The mixture was incubated at room temperature for 3 min, and the absorbance was measured at 734 nm using a spectrophotometer. A Trolox standard curve, ranging from 25 to 1000 µM, was used for quantification. All samples were analyzed in triplicate.

### 2.10. Statistical Analysis

Statistical analysis was performed using SPSS (version 22.0, SPSS Inc., Chicago, IL, USA) on a Windows platform. The data were subjected to analysis of variance followed by Tukey’s range test. A significant difference between the control and treatments was also calculated using the independent sample *t*-test. The difference was considered statistically significant at a *p*-value of less than 0.05.

## 3. Results

### 3.1. Characterization of Antibacterial Substances of Weissella confusa WM36

The antibacterial activity of *W. confusa* WM36-CFS against *S*. Typhimurium remained after catalase and proteinase K treatments but decreased after pH adjustment to 6.0. The untreated LAB CFS, as well as the samples adjusted to pH 4.0 and 5.0, showed clear antibacterial activity, whereas no inhibition was observed at pH 6.0. In addition, catalase- and proteinase K-treated CFS retained antibacterial activity comparable to the untreated control. Sterile MRS broth showed no inhibitory effect (Table 2). These results suggest that acidic metabolites are important contributors to the antibacterial activity of *W. confusa* WM36-CFS, whereas hydrogen peroxide and proteinaceous compounds appear to play a lesser role under the tested conditions. However, the specific organic acids responsible for the observed activity were not quantitatively determined in the present study.

### 3.2. Minimum Effective Concentration of Weissella confusa WM36 Cell-Free Supernatant

The minimum effective concentration (MEC) of WM36-CFS was determined to be 40% (*v*/*v*), as OD600 values at this concentration were comparable to those of the sterile medium control, indicating the absence of detectable bacterial growth. Therefore, a lower concentration of 20% (*v*/*v*) was selected and used as the sub-minimum effective concentration for subsequent experiments.

### 3.3. Effect of Weissella confusa WM36 on Salmonella Typhimurium Growth

The population of *W. confusa* WM36 significantly increased in both monoculture and co-culture with *S. *Typhimurium (*p* < 0.05), reaching concentrations from 7 to 8 log CFU/mL at 12 h in monoculture and at 6 h in co-culture. However, no significant differences were observed between monoculture and co-culture at any time point (*p* > 0.05), as shown in Table 3.

The reduction of *S. *Typhimurium was observed in the presence of viable *W. confusa* and its CFS (Table 4). In the presence of viable *W. confusa*, the population of *S. *Typhimurium decreased from 7 log CFU/mL to below the detectable limit (<1 log CFU/mL), while treatment with CFS resulted in a reduction of *S. *Typhimurium by 1.43 log units within 6 h of treatment. However, there was no *S. *Typhimurium at 12 h of co-culture and CFS treated.

### 3.4. Effect of Weissella confusa WM36 on Salmonella Typhimurium Biofilm Formation

**Figure 1:**
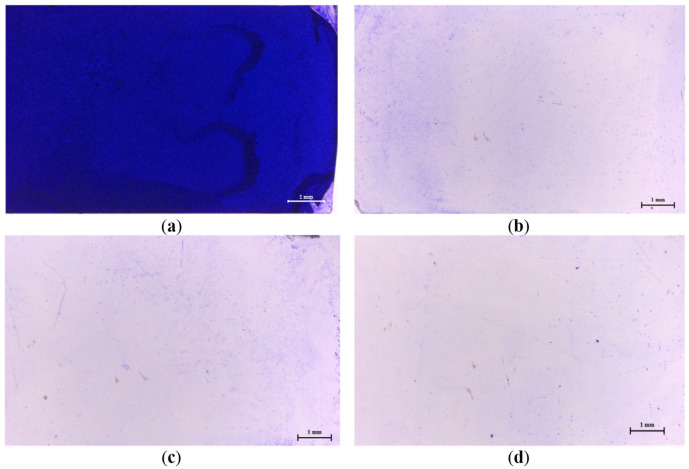
Intensity of biofilm formation attached crystal violet under microscopic visualization (10× magnification) of anti-biofilm activity of *Weissella confusa* WM36 cell-free supernatant against *Salmonella* Typhimurium TISTR 1469. (**a**) Positive control (**b**) 20% (*v*/*v*) (**c**) 40% (*v*/*v*) and (**d**) negative control.

The intensity of *Salmonella* biofilm formation was evaluated. As shown in Figure 1, the CFS at concentrations of 20% (Figure 1b) and 40% (Figure 1c) markedly reduced the visible biofilm accumulation on glass slides compared with the positive control (Figure 1a) and negative control (Figure 1d).

### 3.5. Effect of Weissella confusa WM36 on Salmonella Typhimurium Virulence Gene Expression

In this study, WM36-CFS at 20% (*v*/*v*) directly influenced the expression of *S. *Typhimurium virulence genes. The downregulation of these genes may reduce the virulence-associated behaviors of the bacterium. Genes involved in motility and adhesion, including *filA*, *fimA*, and *flhD*, were significantly downregulated when treated with WM36-CFS. Similarly, genes associated with the T3SS and apparatus, *hilA*, *hilD*, *sopB*, *sopE2*, *sipA*, *sipC, sptP*, and *invF*, were also significantly downregulated (*p* < 0.05) under the same conditions. Moreover, the *luxS* gene, responsible for the autoinducer-2-based QS system, was significantly downregulated (*p* < 0.05), whereas the *sdiA* gene showed an upregulation that was not statistically significant (*p* > 0.05) (Table 5).

### 3.6. Preliminary Safety Evaluation of Weissella confusa WM36

**Figure 2:**
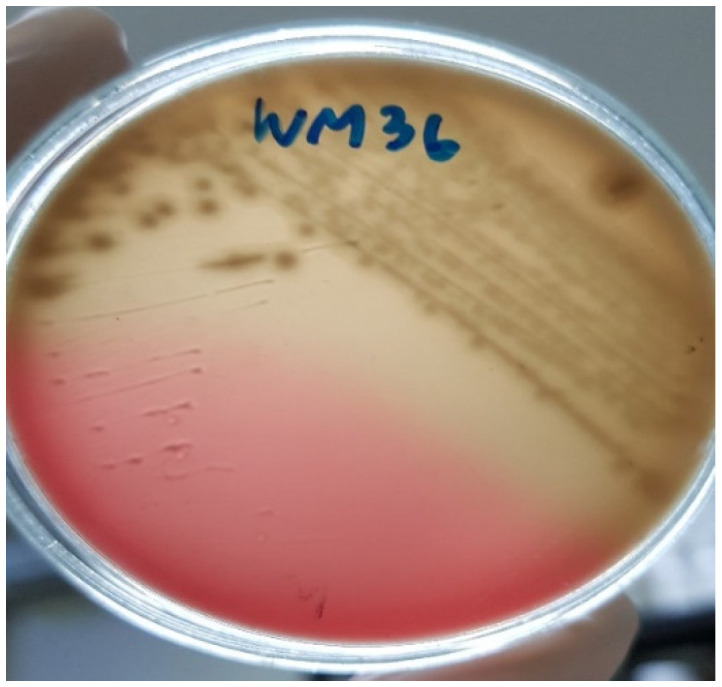
The α-hemolysis produced on blood agar plate by *Weissella confusa* WM36.

The colonies of *W. confusa* WM36 exhibited α-hemolytic activity (Figure 2). Antibiotic susceptibility testing was conducted, and the strain was classified as resistant or sensitive according to the quality control ranges provided by the CLSI guideline. The antibiotic susceptibility profile of WM36 is presented in Table 6. Based on the interpretive criteria applied in this study, *W. confusa* WM36 exhibited reduced susceptibility to ceftriaxone and trimethoprim–sulfamethoxazole. However, these classifications should be interpreted with caution because species-specific breakpoints for *Weissella* spp. are not currently available.

### 3.7. Antioxidant Activities of Weissella confusa WM36 Cell-Free Supernatant

The antioxidant activity of the CFS was evaluated using DPPH and ABTS assays. Results are expressed as Trolox equivalent antioxidant capacity (mg/mL of CFS). The DPPH radical scavenging activity of WM36-CFS was 8.90 ± 0.06 mM Trolox equivalent (Y = 9.2688X − 0.8068; R^2^ = 0.9958), whereas the ABTS assay showed an activity of 13.10 ± 1.42 mM Trolox equivalent (Y = 6.3009X − 9.5877; R^2^ = 0.9807).

## 4. Discussion

*Salmonella* infections cause severe symptoms in hosts and lead to significant economic losses worldwide. Annually, millions of human cases of gastroenteritis and approximately fifteen thousand deaths are attributed to *Salmonella* infections globally. Animals often serve as the main reservoirs for these pathogens, and *Salmonella* is commonly referenced in zoonosis control legislation [35,36,37,38]. Consequently, various strategies have been developed to control *Salmonella*, including the use of beneficial microflora such as LAB, which exhibit antagonistic effects against pathogens. The use of LAB offers multiple benefits without adverse effects on the host. *W. confusa* WM36 was evaluated for its antagonistic activity against *S. *Typhimurium, a causative agent of salmonellosis. Additionally, the preliminary safety of *W. confusa* WM36 as a beneficial strain was assessed, and its CFS was further investigated for antioxidant properties.

In our previous study, *W. confusa* WM36, isolated from fermented grapes in northern Thailand [17], demonstrated strong antagonistic activity against foodborne *Salmonella*. Chemical analysis of the CFS identified organic acids, particularly lactic and acetic acids, together with 2,4-DTBP, as major metabolites associated with antimicrobial activity [18]. In the present study, further characterization of the antibacterial substances produced by *W. confusa* WM36 showed that the antibacterial activity of the CFS remained after catalase and proteinase K treatments but was lost following pH adjustment to 6.0, while activity was retained at pH 4.0 and 5.0. These findings suggest that acidic metabolites contribute substantially to the antibacterial activity observed against *S. *Typhimurium, whereas hydrogen peroxide and proteinaceous compounds appear to play a lesser role under the tested conditions. However, the present study did not quantify individual organic acids or comprehensively characterize all metabolites present in the CFS. Therefore, although previous evidence and the current findings support the involvement of organic acids and 2,4-DTBP in the antagonistic activity of WM36-CFS, the contribution of additional metabolites cannot be excluded. Furthermore, some of these metabolites may also contribute to the anti-biofilm, anti-virulence, and antioxidant activities observed in this study. Future investigations employing comprehensive metabolomic approaches, such as LC-MS/MS, GC-MS, targeted HPLC-based quantification of organic acids, and bioactivity-guided fractionation, are warranted to identify and quantify the full spectrum of active compounds and clarify their individual mechanisms of action.

In this study, *W. confusa* WM36 and its CFS demonstrated antagonistic effects against the tested *S. *Typhimurium strain. Treatment with viable *W. confusa* cells and CFS resulted in complete elimination of detectable *Salmonella* cells within 6–12 h. The complete inhibition observed during co-culture is likely the result of multiple interacting mechanisms rather than a single inhibitory factor. Previous studies identified organic acids and 2,4-DTBP as major metabolites produced by *W. confusa* WM36 [18], while the present findings demonstrated a strong association between antibacterial activity and acidic conditions. In addition to metabolite-mediated inhibition, competition for nutrients and ecological niches may also have contributed to the suppression of *S. *Typhimurium during co-culture. Because pH dynamics, nutrient availability, and metabolite concentrations were not monitored throughout the co-culture period, the relative contribution of each mechanism could not be determined. Future studies incorporating metabolite quantification, pH monitoring, and nutrient utilization analyses would provide a more comprehensive understanding of the mechanisms underlying the antagonistic activity of *W. confusa* WM36. These findings are consistent with previous reports describing the inhibitory activity of *W. confusa* against pathogenic bacteria. For example, *W. confusa* Cys2-2 isolated from spiral ginger inhibited the growth of *S. enterica* [39], while *W. confusa* AI10 showed antimicrobial activity against *S. aureus* and *E. coli* [40]. Similarly, *W. confusa* strains isolated from giant panda feces exhibited strong inhibitory effects against several bacterial pathogens, including *E. coli* ATCC25922, *Salmonella* spp. SC06, and *S. aureus* BJ216 [41].

To further understand the effects of CFS on virulence behaviors beyond *S.* Typhi, experiments were conducted using *S. *Typhimurium. WM36-CFS markedly reduced visible biofilm accumulation on glass slides (Figure 1b,c). This suggests that CFS may reduce *Salmonella* attachment to surfaces, an initial step in biofilm formation [42]. This observation aligns with the gene expression data; the *fimA* gene, responsible for fimbriae-mediated bacterial colonization [43], was downregulated by 20% (*v*/*v*) CFS treatment, while 40% (*v*/*v*) CFS completely eradicated the bacteria (Table 5), resulting in no biofilm formation. However, the anti-biofilm activity of WM36-CFS was evaluated using crystal violet staining and microscopic observation of biofilm formation on glass surfaces. Although this approach clearly demonstrated reduced biofilm accumulation following CFS treatment, quantitative measurements of biofilm biomass were not performed. Therefore, the observed anti-biofilm effect should be interpreted as qualitative evidence of biofilm inhibition. Future studies employing absorbance-based crystal violet quantification, confocal microscopy, or other quantitative biofilm assays would provide a more comprehensive assessment of the anti-biofilm activity of WM36-CFS.

The CFS also strongly suppressed the expression of motility-related genes (*filA* and *flhD*), suggesting a potential reduction in bacterial motility. Likewise, the downregulation of *fimA* may indicate impaired adhesion capability, while suppression of T3SS-associated genes may reflect reduced virulence potential. However, these interpretations are based on transcriptional responses, and the corresponding phenotypic traits were not directly evaluated in the present study. Furthermore, genes involved in the T3SS apparatus and QS system, critical for bacterial invasion and virulence, were downregulated. The metabolites secreted by *W. confusa* WM36, especially 2,4-DTBP, appear to mediate these effects. Notably, 2,4-DTBP has previously been reported to interfere with quorum-sensing-associated behaviors and may contribute to the suppression of virulence-related gene expression observed in the present study. Similar inhibitory effects of 2,4-DTBP on QS-related behaviors have been reported in other bacteria, such as *Chromobacterium violaceum* and *Pseudomonas aeruginosa* [44]. It is important to note that the present study evaluated transcriptional responses rather than phenotypic outcomes. Therefore, although the observed downregulation of motility-, adhesion-, quorum sensing-, and T3SS-associated genes suggests attenuation of virulence-related functions, direct evidence of altered bacterial behavior was not obtained. Future studies should incorporate phenotypic assays, including motility, adhesion, and host-cell invasion analyses, to validate the functional relevance of these transcriptional changes.

Hemolytic activity is an important parameter in the safety assessment of probiotic and biocontrol candidates because it may indicate the presence of virulence-associated traits. In general, γ-hemolysis is considered the most desirable characteristic, whereas β-hemolysis is regarded as a potential safety concern [45,46]. In the present study, *W. confusa* WM36 exhibited α-hemolytic activity. Although α-hemolysis has been reported in several *Weissella* isolates, including strains recovered from clinical samples [47], this characteristic alone is insufficient to establish the safety of a strain for practical application. Therefore, the hemolytic assay performed in this study should be regarded as a preliminary safety assessment. Additional investigations, including genomic screening for virulence-associated determinants and in vivo safety evaluations, are required before *W. confusa* WM36 can be considered for food, animal, or human health applications.

LAB are widely used as multifunctional agents in food production and are commonly found in human, animal, and plant microbiota. The FAO/WHO recommends antibiotic resistance profiling of probiotic strains to minimize the risk of transferring resistance genes to pathogenic bacteria [48], to ensure probiotics do not contribute to conditions such as endocarditis that require antibiotic treatment [49,50], and to support safe concurrent use of probiotics with antibiotic therapy [51]. LAB often exhibit intrinsic resistance to many antibiotics due to resistance determinants on plasmids or chromosomes. Resistance mechanisms can be passive (intrinsic) or active (acquired) [52]. Passive resistance, characterized by modifications of target sites or decreased antibiotic uptake, is generally confined within bacterial clones and has low potential for horizontal gene transfer, posing minimal risk. In contrast, active resistance involves enzymatic alteration or degradation of antibiotics or efflux pumps, facilitating gene transfer across species, and represents a significant public health concern per WHO guidelines [53,54]. The European Commission prohibits LAB strains harboring extrinsic resistance genes from use in food due to potential health risks [55,56].

Previous reports indicate that clinical *W. confusa* isolates exhibit high resistance to vancomycin (>256 µg/mL) [57], while remaining sensitive to several other antibiotics such as penicillin and erythromycin. Other strains from human feces have shown resistance to kanamycin and streptomycin but sensitivity to ampicillin and tetracycline [58]. Additionally, resistance to ceftazidime, cotrimoxazole, metronidazole, rifampin, teicoplanin, and trimethoprim/sulfamethoxazole has been documented [59]. Our WM36 isolate exhibited resistance to two tested antibiotics. To further strengthen the safety assessment of *W. confusa* WM36, molecular characterization of antibiotic resistance determinants is required. Although the present study provides preliminary phenotypic information regarding antibiotic susceptibility, it does not allow discrimination between intrinsic and potentially transferable resistance mechanisms. Therefore, molecular screening approaches, including PCR-based detection of resistance genes and, preferably, whole-genome sequencing (WGS), should be conducted to identify resistance determinants and assess their potential mobility. Such investigations are particularly important before practical applications of this strain in food, animal, or human health settings can be considered, in accordance with current FAO/WHO and EFSA recommendations.

The antioxidant activity of WM36-CFS was evaluated because previous studies identified 2,4-DTBP as a major metabolite produced by *W. confusa* WM36 [18], and this compound has been reported to possess antioxidant properties. Based on these findings, we hypothesized that WM36-CFS may contain bioactive metabolites capable of scavenging free radicals. Therefore, DPPH and ABTS assays were employed as complementary methods to assess its antioxidant potential. The results obtained from both assays confirmed the presence of metabolites with free radical scavenging activity in vitro. Although direct comparisons among studies should be interpreted cautiously because of differences in assay conditions, culture media, metabolite composition, and reporting units, antioxidant activities have also been reported for several LAB-derived metabolites and CFS. Among the metabolites previously identified in WM36-CFS, 2,4-DTBP is likely to contribute to the observed antioxidant activity. Previous studies have shown that 2,4-DTBP exhibits antioxidant activity with IC50 values of 8.2 µM in copper-mediated oxidation, 9.9 µM in AAPH-mediated oxidation, and 52% inhibition in SIN-1-mediated oxidation assays [60]. Similarly, 2,4-DTBP isolated from Lactococcus sp. BSN 307 demonstrated 77.5% free radical scavenging activity in a DPPH assay [61]. The antioxidant capacity observed in WM36-CFS therefore supports the presence of bioactive metabolites with potential functional value. In addition, LAB-derived metabolites may contribute to the alleviation of oxidative stress by directly scavenging reactive oxygen species and by influencing host antioxidant defense systems [41]. Although such effects were not evaluated in the present study, they highlight the potential biological relevance of the antioxidant activity observed in WM36-CFS.

Although the antimicrobial and antioxidant activities of WM36-CFS were evaluated independently in this study, these biological properties may not be entirely unrelated. Previous studies have suggested that certain microbial metabolites, including organic acids and phenolic compounds such as 2,4-DTBP, can exert multiple biological functions, including antimicrobial, anti-biofilm, anti-quorum sensing, and antioxidant activities. Therefore, it is plausible that some of the metabolites produced by *W. confusa* WM36 contribute simultaneously to both the antagonistic effects against *S. *Typhimurium and the antioxidant capacity observed in the CFS. However, the present study does not provide direct evidence of such mechanistic interactions, and future metabolomic and bioactivity-guided investigations are required to identify the specific compounds responsible and to determine whether these activities act synergistically.

## 5. Conclusions

This study demonstrated the antagonistic activity of *W. confusa* WM36 against *S. *Typhimurium. Specifically, both viable *W. confusa* WM36 cells and its CFS inhibited bacterial growth, impaired biofilm formation, and modulated the expression of virulence-associated genes. Preliminary safety assessments provided initial information regarding the safety-related characteristics of *W. confusa* WM36. However, comprehensive safety evaluation remains necessary, including WGS or molecular screening of virulence- and antibiotic-resistance-associated determinants, as well as in vivo safety assessments, before practical applications can be considered. Furthermore, WM36-CFS exhibited antioxidant activity, suggesting that its bioactive metabolites possess multifunctional properties. Collectively, these findings highlight the potential of *W. confusa* WM36 and its CFS as promising sources of antimicrobial, anti-virulence, and antioxidant metabolites for future biocontrol and functional applications against *Salmonella*. Nevertheless, further mechanistic and safety investigations are required to fully characterize the active compounds involved and to validate the suitability of this strain for practical use.

## Figures and Tables

**Table 1:** Lists of forward and reverse primers used in this study.

Genes	Primers (5′–3′)	Amplicon Size (bps)	References
Housekeeping gene			
*16S rRNA*	F: CAGAAGAAGCACCGGCTAACTC R: GCGCTTTACGCCCAGTAATT	87	[29]
Virulence genes			
*filA*	F: CCGCTGAAGGTGTAATGGAT R: CCGCATTTAATAACCCGATG	150	[28]
*fimA*	F: TGTGCCGTCAGCACTAAATC R: TGGTGTTATCTGCCTGACCA	181	This study
*flhD*	F: CAACGAAGAGATGGCAAACA R: GACGCGTTGAAAGCATGATA	185	[28]
*hilA*	F: CATGGCTGGTCAGTTGGAG R: CGTAATTCATCGCCTAAACG	150	[29]
*hilD*	F: ACTCGAGATACCGACGCAAC R: CTTCTGGCAGGAAAGTCAGG	129	[29]
*sopB*	F: AACCGTTCTGGGTAAACAAGAC R: GGTCCGCTTTAACTTTGGCTAAC	77	[29]
*sopE2*	F: GCCTGCATCAACAAACAGACA R: ATACCGCCCTACCCTCAGAAG	72	[29]
*sipA*	F: GGCTTGCGTGCGGAAATA R: ATCGCTACATTGCGCTTTCA	69	[29]
*sipC*	F: CTGTGGCTTTCAGTGGTCAG R: TGCGTTGTCCGGTAGTATTTC	150	[29]
*sptP*	F: ATGCTCGTGCCTGGTGGTGTTA R: ACGGTAACGGCTGGTGATCT	236	[29]
*invF*	F: GCAGGATTAGTGGACACGAC R: TTTACGATCTTGCCAAATAGCG	87	[28]
*sdiA*	F: AGCAGTTTACGCTGCTCCTC R: GCCGTCCACTTCAGAATCTC	164	[18]
*luxS*	F: AGCATCTGTTTGCTGGCTTT R: TCCTGCACTTTCAGCACATC	178	[18]

**Table 2:** Effects of pH, catalase, and proteinase K treatments on the antibacterial activity of *Weissella confusa* WM36-CFS against *Salmonella *Typhimurium.

Treatment	Result
Untreated CFS	+
CFS adjusted to pH 4.0	+
CFS adjusted to pH 5.0	+
CFS adjusted to pH 6.0	−
Catalase-treated CFS	+
Proteinase K-treated CFS	+
Sterile MRS broth	−

Note: +, inhibition; −, no inhibition.

**Table 3:** The count of *Weissella confusa* WM36 during co-cultured with *Salmonella *Typhimurium.

Time (h)	The Count of *Weissella confusa* WM36 (log CFU/mL)	
	Monoculture	Co-Culture
0	7.00 ± 0.11 ^a^	7.09 ± 0.07 ^a^
6	7.85 ± 0.03 ^b^	8.17 ± 0.15 ^b^
12	8.22 ± 0.22 ^b^	8.11 ± 0.05 ^b^
18	8.26 ± 0.07 ^b^	8.09 ± 0.15 ^b^
24	8.04 ± 0.04 ^b^	8.15 ± 0.04 ^b^

All values provided as mean ± standard deviation of triplicate. Values in the same column sharing different superscript letters are significantly different (*p* < 0.05).

**Table 4:** The count of *Salmonella *Typhimurium during co-cultured with *Weissella confusa* WM36 and its cell-free supernatant.

Time (h)	The Count of *Salmonella *Typhimurium (log CFU/mL)		
	Monoculture	Co-Culture	Growth with CFS
0	7.02 ± 0.07 ^a^	7.11 ± 0.07	7.10 ± 0.44 ^a^
6	8.06 ± 0.03 ^b^	ND	5.67 ± 0.11 ^b^
12	8.27 ± 0.17 ^b^	ND	ND
18	8.34 ± 0.18 ^b^	ND	ND
24	8.50 ± 0.15 ^c^	ND	ND

All values are presented as the mean ± standard deviation of triplicate measurements. Values within the same column sharing different superscript letters are significantly different (*p* < 0.05). Values below the detection limit were recorded as non-detectable (ND). Statistical comparisons were not performed for treatments in which *Salmonella* counts were below the detection limit.

**Table 5:** Fold change of *Salmonella* virulence gene expression in the presence of *Weissella confusa* WM36 CFS (20% *v*/*v*).

Genes	Fold Change ^1^	
	Control	WM36-CFS
*filA*	1	0.51 ± 0.01 *
*fimA*	1	0.24 ± 0.08 *
*flhD*	1	0.11 ± 0.04 *
*hilA*	1	0.14 ± 0.01 *
*hilD*	1	0.10 ± 0.03 *
*sopB*	1	0.16 ± 0.01 *
*sopE2*	1	0.06 ± 0.03 *
*sipA*	1	0.08 ± 0.00 *
*sipC*	1	0.05 ± 0.01 *
*sptP*	1	0.08 ± 0.03 *
*invF*	1	0.09 ± 0.00 *
*sdiA*	1	1.11 ± 0.22
*luxS*	1	0.21 ± 0.00 *

^1^ All values are expressed as the mean ± standard deviation of triplicate measurements. The fold change in virulence gene expression was analyzed using the comparative Ct (2^−∆∆Ct^) method. The values obtained from 2^−∆∆Ct^ represent the fold change in gene expression of the sample relative to the reference (control) sample, which has a 2^−∆∆Ct^ value of 1. A fold change value of less than 1 indicates downregulation of gene expression, whereas a value greater than 1 indicates upregulation. Fold change values for each gene were used to compare expression levels between treatments and the control. The asterisk (*) indicates a statistically significant difference (*p* < 0.05) compared with the untreated control.

**Table 6:** The antibiotic susceptibility profile of *Weissella confusa* WM36.

Antibiotics	Clear Zone (mm)/Interpretation
Ampicillin	38 (S)
Azithromycin	22 (S)
Ceftriaxone	24 (R)
Chloramphenicol	30 (S)
Doxycycline	36 (S)
Erythromycin	32 (S)
Levofloxacin	27 (S)
Moxifloxacin	32 (S)
Penicillin	27 (S)
Tetracycline	29 (S)
Trimethoprim–sulfamethoxazole	17 (R)

R: resistant; S: susceptible.

## Data Availability

The original contributions presented in this study are included in the article. Further inquiries can be directed to the first and corresponding author.
